# 2-Amino-4-(4-meth­oxy­phen­yl)-5-oxo-5,6,7,8-tetra­hydro-4*H*-chromene-3-carbonitrile 1,4-dioxane hemisolvate

**DOI:** 10.1107/S1600536812027729

**Published:** 2012-06-23

**Authors:** Shaaban K. Mohamed, Mehmet Akkurt, Muhammad N. Tahir, Antar A. Abdelhamid, Sabry H. H. Younes

**Affiliations:** a Chemistry and Environmental Division, Manchester Metropolitan University, Manchester M1 5GD, England; b Department of Physics, Faculty of Sciences, Erciyes University, 38039 Kayseri, Turkey; c University of Sargodha, Department of Physics, Sargodha, Pakistan; d Chemistry Department, Faculty of Science, Sohag University, Sohag 82524, Egypt

## Abstract

In the crystal structure of the title compound, C_17_H_16_N_2_O_3_·0.5C_4_H_8_O_2_, pairs of N—H⋯N hydrogen bonds link mol­ecules into dimers with *R*_2_^2^(12) motifs, which are connected by N—H⋯O hydrogen bonds, forming a supra­molecular array in the *ab* plane. The 1,4-dioxane ring, which lies about an inversion center, adopts a chair conformation.

## Related literature

For the biological activity of pyran and fused-pyran mol­ecules, see: Bargagna *et al.* (1992 ▶); Symeonidis *et al.* (2009 ▶); Narender & Gupta (2009 ▶); Alvey *et al.* (2009 ▶); Gorlitzer *et al.* (1984 ▶); Han *et al.* (2008 ▶); Martinez & Marco (1997 ▶); Smith *et al.* (1998 ▶); Taylor *et al.* (1998 ▶). For related structures, see: Gourdeau *et al.* (2004 ▶); Foroumadi *et al.* (2007 ▶); Mohamed *et al.* (2012 ▶). For puckering parameters, see: Cremer & Pople (1975 ▶). For hydrogen-bond motifs, see: Bernstein *et al.* (1995 ▶).
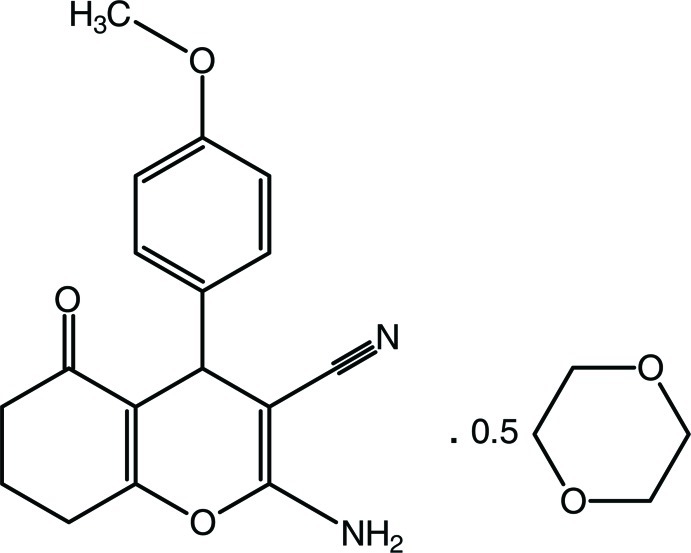


## Experimental

### 

#### Crystal data

C_17_H_16_N_2_O_3_·0.5C_4_H_8_O_2_
*M* *_r_* = 340.37
Triclinic, 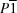

*a* = 8.0876 (4) Å
*b* = 9.2013 (4) Å
*c* = 12.1613 (6) Å
α = 94.376 (2)°
β = 102.827 (1)°
γ = 95.972 (2)°
*V* = 873.01 (7) Å^3^
*Z* = 2
Mo *K*α radiation
μ = 0.09 mm^−1^
*T* = 293 K
0.35 × 0.25 × 0.22 mm

#### Data collection

Bruker Kappa APEXII CCD diffractometer
Absorption correction: multi-scan (*SADABS*; Bruker, 2005 ▶) *T*_min_ = 0.973, *T*_max_ = 0.980
14186 measured reflections
4108 independent reflections
3134 reflections with *I* > 2σ(*I*)
*R*_int_ = 0.021

#### Refinement

*R*[*F* ^2^ > 2σ(*F* ^2^)] = 0.059
*wR*(*F* ^2^) = 0.194
*S* = 1.07
4108 reflections
227 parameters
H-atom parameters constrained
Δρ_max_ = 0.60 e Å^−3^
Δρ_min_ = −0.39 e Å^−3^

### 

Data collection: *APEX2* (Bruker, 2007 ▶); cell refinement: *SAINT* (Bruker, 2007 ▶); data reduction: *SAINT*; program(s) used to solve structure: *SHELXS97* (Sheldrick, 2008 ▶); program(s) used to refine structure: *SHELXL97* (Sheldrick, 2008 ▶); molecular graphics: *ORTEP-3 for Windows* (Farrugia, 1997 ▶) and *PLATON* (Spek, 2009 ▶); software used to prepare material for publication: *WinGX* (Farrugia, 1999 ▶) and *PLATON*.

## Supplementary Material

Crystal structure: contains datablock(s) global, I. DOI: 10.1107/S1600536812027729/tk5115sup1.cif

Structure factors: contains datablock(s) I. DOI: 10.1107/S1600536812027729/tk5115Isup2.hkl

Supplementary material file. DOI: 10.1107/S1600536812027729/tk5115Isup3.cml

Additional supplementary materials:  crystallographic information; 3D view; checkCIF report

## Figures and Tables

**Table 1:** Hydrogen-bond geometry (Å, °)

*D*—H⋯*A*	*D*—H	H⋯*A*	*D*⋯*A*	*D*—H⋯*A*
N1—H1*A*⋯N2^i^	0.86	2.27	3.123 (3)	171
N1—H1*B*⋯O3^ii^	0.86	2.10	2.945 (2)	167

Symmetry codes: (i) 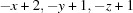; (ii) 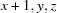.

**Fig. 1.**
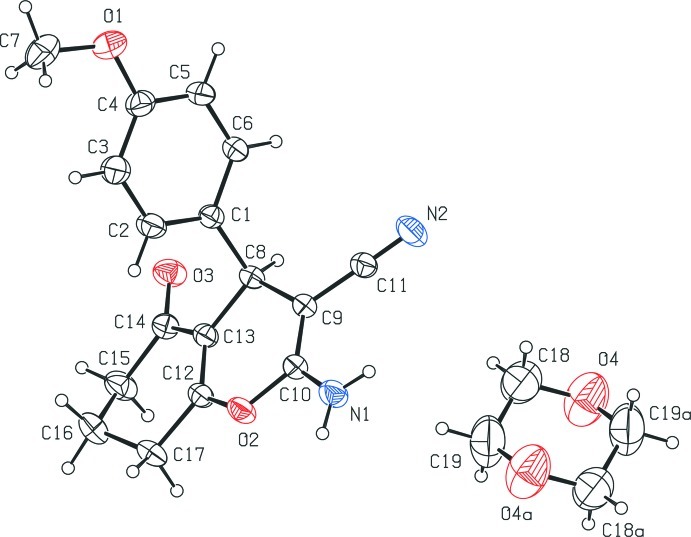
View of the title compound with the atom numbering scheme. Displacement ellipsoids for non-H atoms are drawn at the 30% probability level.

**Fig. 2.**
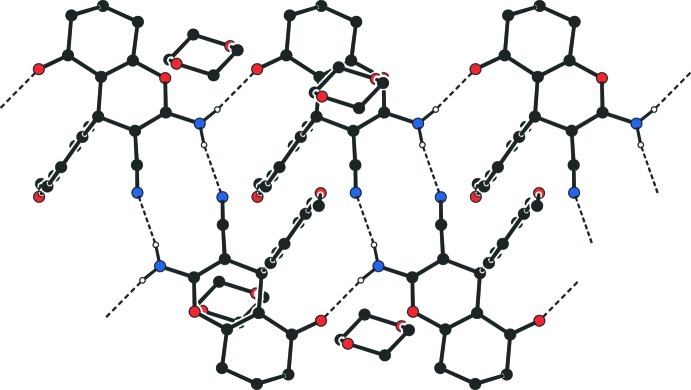
View of the dimers formed by pairs of N—H···N hydrogen bonds (dashed lines), with an R22(12) motif, and the N—H···O hydrogen bonds (dashed lines) which connect the dimers with each other, forming a two-dimensional array. H atoms not involved in hydrogen bonding have been omitted for clarity. [Symmetry code: (a) 2 - x, 2 - y, 2 - z].

## supplementary crystallographic information

### Comment

Pyran and fused pyran ring systems are biologically interesting compounds known for their antimicrobial and antifungal (Alvey, *et al.*, 2009), antioxidant (Symeonidis *et al.*, 2009), antileishmanial (Narender *et al.*, 2009), antitumor (Han *et al.*, 2008). In addition, fused chromene ring systems have platelet antiaggregating, local anesthetic (Bargagna *et al.* 1992) and antihistaminic activities (Gorlitzer *et al.* 1984). They also exhibit inhibitory effects on influenza virus sialidases (Smith *et al.* 1998; Taylor *et al.* 1998) and antiviral activities (Martinez & Marco, 1997). Such observations prompted us to report the synthesis and crystal structure of the title compound (I).

In (I), Fig. 1, the O2/C8—C10/C12/C13 4*H*-pyran and C12–C17 cyclohexene rings are puckered with puckering parameters (Cremer & Pople, 1975) of Q_T_ = 0.187 (2) Å, θ = 72.2 (5) °, φ = 175.7 (6) ° and Q_T_ = 0.455 (2) ° A, θ = 122.9 (3) °, φ = 48.5 (3) °, respectively. The centroid of the solvent 1,4-dioxane ring (O4/C18/C19/O4a/C18*a*/C19*a*) lies about an inversion center. The 1,4-dioxane ring adopts a chair conformation [puckering parameters Q_T_ = 0.560 (5) Å, θ = 3.46 (3) °, φ = 0.00 °]. The values of the bond lengths and angles in (I) are in normal ranges and are comparable with those of related structures (Gourdeau *et al.*, 2004; Foroumadi *et al.*, 2007; Mohamed *et al.*, 2012).

In the crystal, molecules are linked by the pairs of N—H···N hydrogen bonds, forming dimers, with an *R*^2^_2_(12) motif (Bernstein *et al.*, 1995; Table 1, Fig. 2). These dimers are connected through the N—H···O hydrogen bonds with each other (Table 1, Fig. 2).

### Experimental

A mixture of (4-methoxybenzylidene)propanedinitrile (184 mg, 1 mmol), cyclohexane-1,3-dione (112 mg, 1 mmol) in presence of ethanolamine (61 mg) as catalyst was refluxed in ethanol (50 ml). The reaction mixture was monitored by TLC until completion after 7 h. A solid product was deposited on cooling at ambient temperature and collected by filtration. The crude product was washed with dioxane and recrystallized from ethanol/drops of dioxane to afford the title compound in 78% yield. Single crystals suitable for X-ray analysis were grown up on slow evaporation of its mixed solvent ethanol/dioxane (9:1) solution at room temperature over three days. *M*.pt: 435 K.

### Refinement

All H atoms were positioned geometrically and refined by using a riding model, with N—H = 0.86 Å and C—H = 0.93 Å (aromatic), 0.96 Å (methyl), 0.97 Å (methylene) and 0.98 Å (methine), with *U*_iso_(H) = 1.5*U*_eq_(C) for methyl-H and *U*_iso_(H) = 1.2*U*_eq_(C, N) for other H-atoms.

### Crystal data

**Table:**

C_17_H_16_N_2_O_3_·0.5C_4_H_8_O_2_	*Z* = 2
*M**_r_* = 340.37	*F*(000) = 360
Triclinic, *P*1	*D*_x_ = 1.295 Mg m^−^^3^
Hall symbol: -P 1	Mo *K*α radiation, λ = 0.71073 Å
*a* = 8.0876 (4) Å	Cell parameters from 420 reflections
*b* = 9.2013 (4) Å	θ = 3.6–22.5°
*c* = 12.1613 (6) Å	µ = 0.09 mm^−^^1^
α = 94.376 (2)°	*T* = 293 K
β = 102.827 (1)°	Prism, light-yellow
γ = 95.972 (2)°	0.35 × 0.25 × 0.22 mm
*V* = 873.01 (7) Å^3^	

### Data collection

**Table:**

Bruker Kappa APEXII CCD diffractometer	4108 independent reflections
Radiation source: fine-focus sealed tube	3134 reflections with *I* > 2σ(*I*)
Graphite monochromator	*R*_int_ = 0.021
Detector resolution: 0.81 pixels mm^-1^	θ_max_ = 27.9°, θ_min_ = 1.7°
ω scans	*h* = −10→10
Absorption correction: multi-scan (*SADABS*; Bruker, 2005)	*k* = −12→8
*T*_min_ = 0.973, *T*_max_ = 0.980	*l* = −16→15
14186 measured reflections	

### Refinement

**Table:**

Refinement on *F*^2^	Primary atom site location: structure-invariant direct methods
Least-squares matrix: full	Secondary atom site location: difference Fourier map
*R*[*F*^2^ > 2σ(*F*^2^)] = 0.059	Hydrogen site location: inferred from neighbouring sites
*wR*(*F*^2^) = 0.194	H-atom parameters constrained
*S* = 1.07	*w* = 1/[σ^2^(*F*_o_^2^) + (0.0997*P*)^2^ + 0.273*P*] where *P* = (*F*_o_^2^ + 2*F*_c_^2^)/3
4108 reflections	(Δ/σ)_max_ < 0.001
227 parameters	Δρ_max_ = 0.60 e Å^−^^3^
0 restraints	Δρ_min_ = −0.39 e Å^−^^3^

### Special details

**Table:**

Geometry. Bond distances, angles *etc*. have been calculated using the rounded fractional coordinates. All su's are estimated from the variances of the (full) variance-covariance matrix. The cell e.s.d.'s are taken into account in the estimation of distances, angles and torsion angles
Refinement. Refinement on *F*^2^ for ALL reflections except those flagged by the user for potential systematic errors. Weighted *R*-factors *wR* and all goodnesses of fit *S* are based on *F*^2^, conventional *R*-factors *R* are based on *F*, with *F* set to zero for negative *F*^2^. The observed criterion of *F*^2^ > σ(*F*^2^) is used only for calculating -*R*-factor-obs *etc*. and is not relevant to the choice of reflections for refinement. *R*-factors based on *F*^2^ are statistically about twice as large as those based on *F*, and *R*-factors based on ALL data will be even larger.

### Fractional atomic coordinates and isotropic or equivalent isotropic displacement parameters (Å^2^)

**Table:**

	*x*	*y*	*z*	*U*_iso_*/*U*_eq_	
O1	0.1813 (2)	0.42680 (19)	0.00491 (15)	0.0760 (6)	
O2	0.93204 (16)	0.95097 (14)	0.33842 (12)	0.0512 (4)	
O3	0.36871 (18)	0.99158 (15)	0.37001 (14)	0.0595 (5)	
N1	1.0877 (2)	0.77420 (19)	0.39337 (15)	0.0563 (6)	
N2	0.8057 (3)	0.5088 (2)	0.49805 (19)	0.0712 (7)	
C1	0.5009 (2)	0.70436 (17)	0.26984 (14)	0.0389 (5)	
C2	0.4984 (3)	0.7302 (2)	0.15944 (17)	0.0531 (6)	
C3	0.3939 (3)	0.6405 (2)	0.06812 (18)	0.0583 (7)	
C4	0.2893 (3)	0.5219 (2)	0.08782 (18)	0.0537 (6)	
C5	0.2917 (3)	0.4941 (2)	0.19793 (19)	0.0563 (7)	
C6	0.3951 (2)	0.58441 (19)	0.28771 (17)	0.0476 (6)	
C7	0.1568 (4)	0.4634 (3)	−0.1082 (2)	0.0809 (9)	
C8	0.6180 (2)	0.79962 (17)	0.37058 (14)	0.0387 (5)	
C9	0.7912 (2)	0.74660 (18)	0.40311 (14)	0.0405 (5)	
C10	0.9333 (2)	0.81758 (19)	0.38054 (14)	0.0420 (5)	
C11	0.8019 (2)	0.6148 (2)	0.45477 (17)	0.0487 (6)	
C12	0.7895 (2)	1.02140 (18)	0.33096 (14)	0.0422 (5)	
C13	0.6429 (2)	0.95783 (18)	0.34927 (14)	0.0403 (5)	
C14	0.5022 (2)	1.04491 (19)	0.35100 (15)	0.0461 (6)	
C15	0.5308 (3)	1.2042 (2)	0.3328 (2)	0.0634 (8)	
C16	0.6528 (3)	1.2323 (2)	0.2563 (2)	0.0660 (8)	
C17	0.8203 (3)	1.1737 (2)	0.29963 (18)	0.0537 (6)	
O4	0.8992 (4)	0.9746 (4)	1.0758 (2)	0.1475 (16)	
C18	0.8279 (5)	0.9756 (5)	0.9615 (4)	0.1243 (19)	
C19	0.9322 (6)	1.0707 (6)	0.9113 (4)	0.138 (2)	
H1A	1.10620	0.69140	0.41900	0.0680*	
H1B	1.16900	0.82900	0.37610	0.0680*	
H2	0.56870	0.81000	0.14570	0.0640*	
H3	0.39450	0.66020	−0.00570	0.0700*	
H5	0.22270	0.41340	0.21170	0.0680*	
H6	0.39390	0.56470	0.36140	0.0570*	
H7A	0.11860	0.55860	−0.11240	0.1210*	
H7B	0.07240	0.39170	−0.15710	0.1210*	
H7C	0.26270	0.46480	−0.13150	0.1210*	
H8	0.56480	0.79380	0.43530	0.0460*	
H15A	0.57660	1.26170	0.40550	0.0760*	
H15B	0.42210	1.23650	0.29930	0.0760*	
H16A	0.60040	1.18570	0.18050	0.0790*	
H16B	0.67420	1.33710	0.25170	0.0790*	
H17A	0.88530	1.17380	0.24150	0.0640*	
H17B	0.88670	1.23700	0.36540	0.0640*	
H18A	0.71550	1.00780	0.95170	0.1500*	
H18B	0.81450	0.87690	0.92390	0.1500*	
H19A	0.94160	1.17020	0.94680	0.1660*	
H19B	0.87970	1.07000	0.83140	0.1660*	

### Atomic displacement parameters (Å^2^)

**Table:**

	*U*^11^	*U*^22^	*U*^33^	*U*^12^	*U*^13^	*U*^23^
O1	0.0761 (11)	0.0640 (10)	0.0702 (10)	−0.0133 (8)	−0.0058 (8)	−0.0056 (8)
O2	0.0475 (7)	0.0467 (7)	0.0657 (8)	0.0030 (5)	0.0229 (6)	0.0207 (6)
O3	0.0480 (8)	0.0506 (8)	0.0832 (10)	0.0060 (6)	0.0217 (7)	0.0084 (7)
N1	0.0429 (9)	0.0552 (10)	0.0748 (11)	0.0057 (7)	0.0169 (8)	0.0229 (8)
N2	0.0600 (11)	0.0595 (11)	0.1025 (16)	0.0096 (8)	0.0244 (10)	0.0413 (11)
C1	0.0359 (8)	0.0347 (8)	0.0472 (9)	0.0047 (6)	0.0112 (7)	0.0063 (6)
C2	0.0594 (12)	0.0473 (10)	0.0514 (10)	−0.0082 (8)	0.0170 (9)	0.0062 (8)
C3	0.0649 (13)	0.0591 (12)	0.0475 (10)	−0.0043 (10)	0.0117 (9)	0.0042 (9)
C4	0.0487 (11)	0.0441 (10)	0.0612 (12)	0.0022 (8)	0.0016 (9)	−0.0009 (8)
C5	0.0505 (11)	0.0417 (10)	0.0702 (13)	−0.0070 (8)	0.0040 (9)	0.0124 (9)
C6	0.0454 (10)	0.0423 (9)	0.0543 (10)	0.0002 (7)	0.0091 (8)	0.0148 (8)
C7	0.0762 (17)	0.0896 (18)	0.0618 (14)	0.0016 (13)	−0.0056 (12)	−0.0107 (13)
C8	0.0407 (8)	0.0354 (8)	0.0418 (8)	0.0004 (6)	0.0145 (7)	0.0071 (6)
C9	0.0423 (9)	0.0372 (8)	0.0413 (8)	0.0004 (6)	0.0085 (7)	0.0087 (6)
C10	0.0443 (9)	0.0398 (8)	0.0414 (8)	0.0015 (7)	0.0092 (7)	0.0082 (7)
C11	0.0413 (9)	0.0462 (10)	0.0586 (11)	0.0016 (7)	0.0104 (8)	0.0144 (8)
C12	0.0499 (10)	0.0354 (8)	0.0420 (9)	0.0005 (7)	0.0133 (7)	0.0070 (7)
C13	0.0460 (9)	0.0341 (8)	0.0407 (8)	0.0008 (7)	0.0114 (7)	0.0049 (6)
C14	0.0494 (10)	0.0393 (9)	0.0481 (10)	0.0031 (7)	0.0091 (8)	0.0041 (7)
C15	0.0664 (13)	0.0403 (10)	0.0882 (16)	0.0107 (9)	0.0234 (12)	0.0150 (10)
C16	0.0820 (16)	0.0462 (11)	0.0748 (14)	0.0089 (10)	0.0219 (12)	0.0245 (10)
C17	0.0659 (12)	0.0391 (9)	0.0605 (11)	−0.0019 (8)	0.0251 (10)	0.0135 (8)
O4	0.125 (2)	0.236 (4)	0.0879 (17)	0.028 (2)	0.0352 (16)	0.018 (2)
C18	0.105 (3)	0.152 (4)	0.107 (3)	0.002 (3)	0.010 (2)	0.020 (3)
C19	0.114 (3)	0.200 (5)	0.111 (3)	0.038 (3)	0.022 (2)	0.069 (3)

### Geometric parameters (Å, º)

**Table:**

O1—C4	1.364 (3)	C13—C14	1.461 (2)
O1—C7	1.417 (3)	C14—C15	1.501 (3)
O2—C10	1.366 (2)	C15—C16	1.516 (3)
O2—C12	1.369 (2)	C16—C17	1.511 (3)
O3—C14	1.216 (2)	C2—H2	0.9300
O4—C18	1.383 (5)	C3—H3	0.9300
O4—C19^i^	1.446 (6)	C5—H5	0.9300
N1—C10	1.330 (2)	C6—H6	0.9300
N2—C11	1.143 (3)	C7—H7C	0.9600
N1—H1B	0.8600	C7—H7A	0.9600
N1—H1A	0.8600	C7—H7B	0.9600
C1—C2	1.377 (3)	C8—H8	0.9800
C1—C8	1.521 (2)	C15—H15B	0.9700
C1—C6	1.386 (2)	C15—H15A	0.9700
C2—C3	1.388 (3)	C16—H16A	0.9700
C3—C4	1.379 (3)	C16—H16B	0.9700
C4—C5	1.379 (3)	C17—H17B	0.9700
C5—C6	1.376 (3)	C17—H17A	0.9700
C8—C13	1.499 (2)	C18—C19	1.417 (7)
C8—C9	1.511 (2)	C18—H18A	0.9700
C9—C10	1.354 (2)	C18—H18B	0.9700
C9—C11	1.410 (3)	C19—H19A	0.9700
C12—C13	1.338 (2)	C19—H19B	0.9700
C12—C17	1.491 (3)		
			
C4—O1—C7	117.78 (19)	C2—C3—H3	120.00
C10—O2—C12	118.91 (14)	C6—C5—H5	120.00
C18—O4—C19^i^	108.6 (3)	C4—C5—H5	120.00
H1A—N1—H1B	120.00	C1—C6—H6	120.00
C10—N1—H1B	120.00	C5—C6—H6	120.00
C10—N1—H1A	120.00	O1—C7—H7A	109.00
C2—C1—C6	117.77 (17)	O1—C7—H7B	109.00
C6—C1—C8	119.84 (15)	H7A—C7—H7B	109.00
C2—C1—C8	122.37 (15)	H7A—C7—H7C	109.00
C1—C2—C3	121.88 (19)	H7B—C7—H7C	110.00
C2—C3—C4	119.41 (19)	O1—C7—H7C	109.00
O1—C4—C5	116.23 (19)	C9—C8—H8	108.00
C3—C4—C5	119.3 (2)	C13—C8—H8	108.00
O1—C4—C3	124.49 (19)	C1—C8—H8	108.00
C4—C5—C6	120.73 (19)	C14—C15—H15B	109.00
C1—C6—C5	120.93 (18)	C16—C15—H15A	109.00
C9—C8—C13	108.63 (14)	C16—C15—H15B	109.00
C1—C8—C13	112.38 (14)	H15A—C15—H15B	108.00
C1—C8—C9	111.95 (13)	C14—C15—H15A	109.00
C8—C9—C10	122.54 (15)	C15—C16—H16B	109.00
C10—C9—C11	119.42 (16)	C17—C16—H16A	109.00
C8—C9—C11	118.00 (14)	C15—C16—H16A	109.00
O2—C10—N1	110.30 (15)	H16A—C16—H16B	108.00
N1—C10—C9	128.22 (17)	C17—C16—H16B	109.00
O2—C10—C9	121.48 (15)	C12—C17—H17A	110.00
N2—C11—C9	177.6 (2)	C12—C17—H17B	110.00
O2—C12—C13	122.91 (15)	C16—C17—H17B	110.00
C13—C12—C17	125.75 (17)	H17A—C17—H17B	108.00
O2—C12—C17	111.34 (16)	C16—C17—H17A	110.00
C8—C13—C14	118.19 (14)	O4—C18—C19	110.9 (4)
C8—C13—C12	122.27 (15)	O4^i^—C19—C18	110.8 (4)
C12—C13—C14	119.54 (15)	O4—C18—H18A	109.00
C13—C14—C15	117.57 (16)	O4—C18—H18B	109.00
O3—C14—C13	121.28 (16)	C19—C18—H18A	109.00
O3—C14—C15	121.11 (17)	C19—C18—H18B	109.00
C14—C15—C16	112.24 (17)	H18A—C18—H18B	108.00
C15—C16—C17	111.57 (18)	C18—C19—H19A	109.00
C12—C17—C16	110.51 (18)	C18—C19—H19B	109.00
C1—C2—H2	119.00	H19A—C19—H19B	108.00
C3—C2—H2	119.00	O4^i^—C19—H19A	109.00
C4—C3—H3	120.00	O4^i^—C19—H19B	110.00
			
C7—O1—C4—C3	−9.3 (3)	C1—C8—C9—C10	105.99 (18)
C7—O1—C4—C5	171.1 (2)	C1—C8—C9—C11	−71.4 (2)
C10—O2—C12—C17	171.59 (15)	C9—C8—C13—C14	−161.46 (15)
C12—O2—C10—N1	−172.95 (15)	C1—C8—C13—C14	74.12 (19)
C10—O2—C12—C13	−8.8 (2)	C9—C8—C13—C12	17.3 (2)
C12—O2—C10—C9	7.4 (2)	C11—C9—C10—N1	5.4 (3)
C18^i^—O4^i^—C19—C18	−58.0 (5)	C8—C9—C10—O2	7.6 (3)
C19^i^—O4—C18—C19	−58.0 (5)	C8—C9—C10—N1	−172.00 (17)
C8—C1—C2—C3	178.46 (19)	C11—C9—C10—O2	−175.05 (16)
C8—C1—C6—C5	−178.09 (17)	C17—C12—C13—C8	174.55 (17)
C2—C1—C6—C5	0.2 (3)	C17—C12—C13—C14	−6.7 (3)
C2—C1—C8—C9	−87.6 (2)	O2—C12—C17—C16	162.26 (16)
C2—C1—C8—C13	34.9 (2)	C13—C12—C17—C16	−17.3 (3)
C6—C1—C8—C9	90.63 (19)	O2—C12—C13—C14	173.82 (15)
C6—C1—C8—C13	−146.80 (16)	O2—C12—C13—C8	−5.0 (3)
C6—C1—C2—C3	0.2 (3)	C12—C13—C14—O3	−178.26 (18)
C1—C2—C3—C4	0.0 (3)	C8—C13—C14—O3	0.6 (3)
C2—C3—C4—C5	−0.5 (3)	C12—C13—C14—C15	−0.5 (3)
C2—C3—C4—O1	179.8 (2)	C8—C13—C14—C15	178.31 (16)
O1—C4—C5—C6	−179.37 (19)	O3—C14—C15—C16	−151.3 (2)
C3—C4—C5—C6	1.0 (3)	C13—C14—C15—C16	31.0 (3)
C4—C5—C6—C1	−0.8 (3)	C14—C15—C16—C17	−54.6 (2)
C13—C8—C9—C11	163.92 (15)	C15—C16—C17—C12	46.9 (2)
C1—C8—C13—C12	−107.09 (18)	O4—C18—C19—O4^i^	59.3 (5)
C13—C8—C9—C10	−18.7 (2)		

Symmetry code: (i) −*x*+2, −*y*+2, −*z*+2.

### Hydrogen-bond geometry (Å, º)

**Table:**

*D*—H···*A*	*D*—H	H···*A*	*D*···*A*	*D*—H···*A*
N1—H1*A*···N2^ii^	0.86	2.27	3.123 (3)	171
N1—H1*B*···O3^iii^	0.86	2.10	2.945 (2)	167

Symmetry codes: (ii) −*x*+2, −*y*+1, −*z*+1; (iii) *x*+1, *y*, *z*.
